# The role of interleukin-6 in diabetic retinal disease: pathophysiology and therapeutic targeting

**DOI:** 10.3389/fimmu.2026.1805665

**Published:** 2026-04-17

**Authors:** Rachael E. A. Harlow, Stefan Rose-John, Zdenka Haskova, Sascha Fauser, Marina Mesquida

**Affiliations:** 1 Roche Pharma Research and Early Development, F. Hoffmann-La Roche Ltd., Basel, Switzerland; 2 Biochemical Institute, Medical Faculty, Christian-Albrechts-University, Kiel, Germany; 3 Genentech, Inc., South San Francisco, CA, United States

**Keywords:** blood-retina barrier, diabetic macular edema, diabetic retinopathy, interleukin-6, ocular inflammation and immunology

## Abstract

Therapeutic strategies in the management of diabetic retinal diseases have typically employed anti–vascular endothelial growth factor A (anti–VEGF-A) therapies. While generally effective, clinical trials and real-world analyses demonstrate that a substantial proportion of patients do not show adequate response to this drug class, with retinal edema persisting in upwards of 60% of cases after one to two years of therapy, exhibiting suboptimal visual outcomes and insufficient disease control, with VEGF independent pathways remaining unaddressed. Inflammation is increasingly recognized as a pivotal pathogenic driver in diabetic eye disease, with Interleukin-6 (IL-6) identified as a central mediator of acute and chronic inflammatory responses. This review discusses the role of inflammation in diabetic retinal disease and synthesizes emerging evidence regarding the therapeutic targeting of IL-6. We highlight the differences between cis-, trans-, and cluster signaling, and describe the IL-6 buffer system. We review preclinical evidence demonstrating how IL-6 signaling disrupts the blood-retinal barrier, both directly and synergistically with VEGF. Finally, we describe the emerging clinical evidence for selective IL-6 and bispecific IL-6/VEGF monoclonal antibodies currently in drug development. These novel approaches aim to address the multiple pathogenic pathways that drive Diabetic Macular Edema (DME), with potential to show superior efficacy.

## 1 Introduction

**Figure 1:**
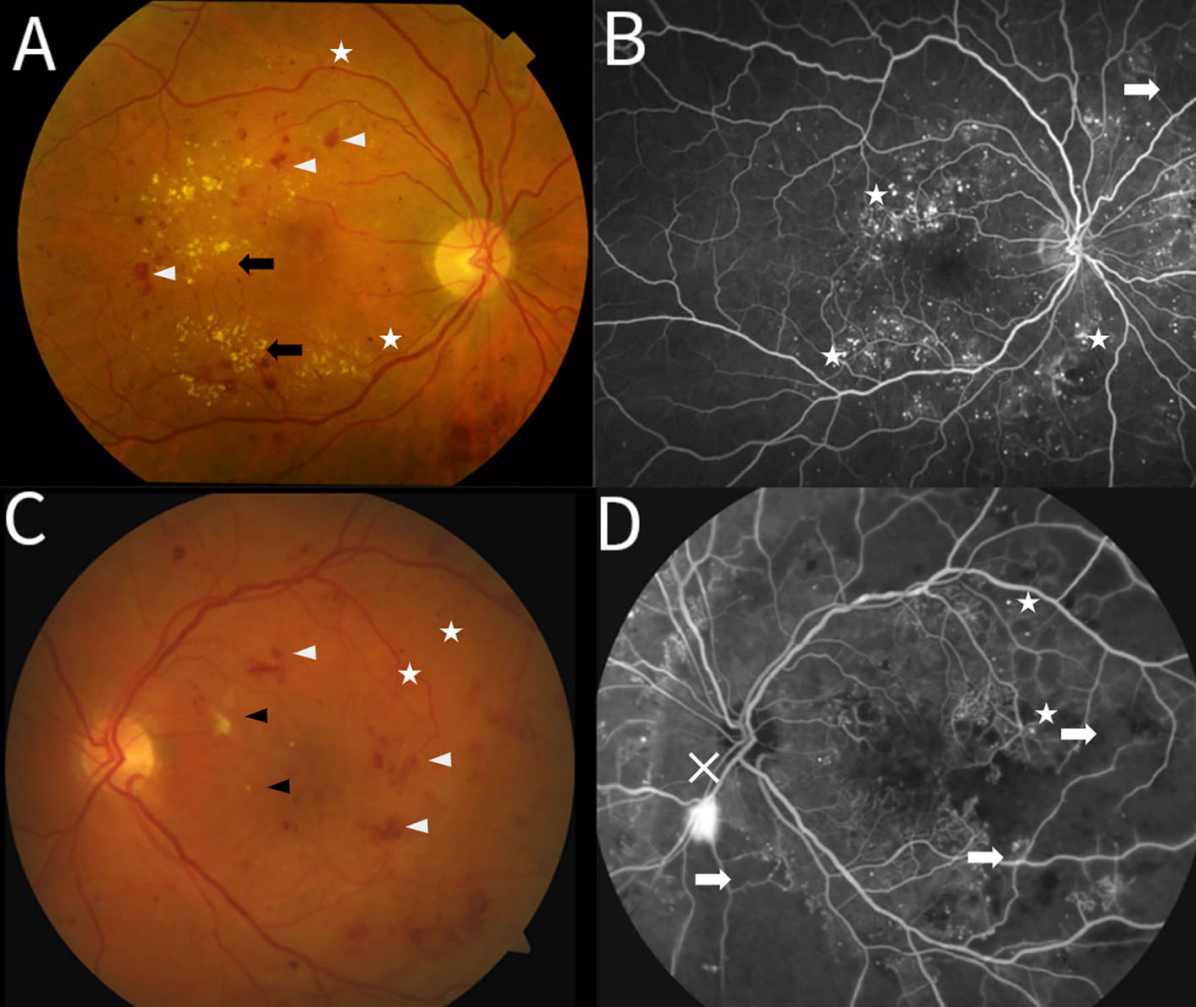
Clinical features of nonproliferative diabetic retinopathy **(A, B)** and proliferative diabetic retinopathy **(C, D)**. **(A)** Color fundus photograph of the right eye displaying scattered intraretinal hemorrhages (white arrowheads), clusters of hard exudates (black arrows) forming a circinate pattern, and microaneurysms (white stars). **(B)** Fluorescein angiogram (FA) of the right eye, highlighting numerous microaneurysms (white stars) as hyperfluorescent pinpoint dots. A localized area of capillary dropout/non-perfusion is visible (white arrow). **(C)** Color fundus photograph of the left eye showing multiple intra retinal hemorrhages (white arrowheads) and microaneurysms (white stars). Black arrowheads are also visible here pointing to pale lesions, representing cotton wool spots. **(D)** Fluorescein angiogram of the left eye revealing extensive areas of capillary non-perfusion (white arrows) presenting as dark, featureless zones. Microaneurysms (white stars) are visible bordering the ischemic areas. Hyperfluorescent leakage at the optic disc indicates active neovascularization (white X).

Diabetic Retinopathy (DR) and diabetic macular edema (DME) represent leading causes of preventable vision loss worldwide, affecting over 100 million people (1). DR is a progressive condition that is clinically staged according to the severity of observable vascular changes in the retina (**Table 1**; **Figure 1**) (2). DME is a severe microvascular complication of diabetes mellitus, characterized by the pathological thickening of the central retina (macula) secondary to the accumulation of intraretinal and/or subretinal fluid in the macular area. This accumulation results from the compromise and breakdown of the inner and outer blood-retinal barrier (BRB) integrity, which is induced by chronic hyperglycemia and its metabolic consequences on the retinal vasculature (3).

**Table 1:** Classification of diabetic retinopathy.

Diabetic retinopathy disease severity level	Observable features on dilated ophthalmoscopy
No apparent retinopathy	No visible abnormalities
NPDR	
Mild NPDR	Microaneurysms only
Moderate NPDR	More than just microaneurysms but less than severe NPDR
Severe NPDR	Any of the following: More than 20 intraretinal hemorrhages in each of the four quadrants Definite venous beading in two or more quadrants Prominent intraretinal microvascular abnormalities in one or more quadrants No signs of PDR
PDR	
PDR	One or more of the following: Vitreous/preretinal hemorrhage Retinal neovascularization in the disk or elsewhere

NPDR, nonproliferative diabetic retinopathy; PDR, proliferative diabetic retinopathy.

The standard of care for DME has evolved significantly since the introduction of the first anti-vascular endothelial growth factor (VEGF) therapy pegaptanib (Macugen) (4), which utilized an RNA aptamer to target VEGF. Since then, the therapeutic landscape has expanded to include several highly effective agents based on monoclonal antibodies and recombinant proteins. Although anti- VEGF therapies have become the standard of care for DME, clinical and real-world data indicate that a significant proportion of patients exhibit suboptimal visual gains and inadequate disease control (5, 6). Real-world analyses demonstrate average visual acuity improvements of only 4.0 to 5.5 letters at 12 months, with patients possessing good baseline vision often experiencing a mean vision loss (6). Furthermore, clinical trial data reveal that persistent macular thickening in eyes with centrally-involving DME remains in 31.6% to 65.6% of eyes through 24 weeks of anti-VEGF therapy, and up to 68.2% of these cases chronically persist through to two years of treatment (5). This insufficient response suggests the presence of unaddressed pathological mechanisms beyond VEGF, highlighting an unmet need for therapies that target the multifactorial nature of the disease (7).

Increasing evidence suggests chronic, low-grade inflammation as a key pathogenic pathway in the development and progression of diabetic retinal diseases (8, 9). This sustained inflammatory state coined as “parainflammation” (10) is driven by metabolic dysregulation and tissue stress, and may explain why a subset of patients remains only partially responsive to anti-VEGF monotherapy (11). The pleiotropic cytokine interleukin-6 (IL-6) plays a pivotal role in orchestrating both acute and chronic inflammatory responses in the retina (12, 13). Intraocular IL-6 concentrations of patients with DR and DME are elevated and correlate with disease severity, progression, and incomplete response to anti-VEGF therapy (14–16). Furthermore, IL-6 contributes to retinal pathophysiology by upregulating VEGF (17), provoking breakdown of the BRB (18, 19), and promoting leukostasis and immune cell infiltration into the retinal tissue (8, 20). As a central cytokine in the inflammatory network, IL-6 represents a promising target for therapeutic intervention in retinal disease. Furthermore, inhibiting IL-6 signaling may address the inflammatory component of the pathophysiology of DR/DME, offering a more holistic management.

## 2 Diabetic retinopathy pathogenesis

**Figure 2:**
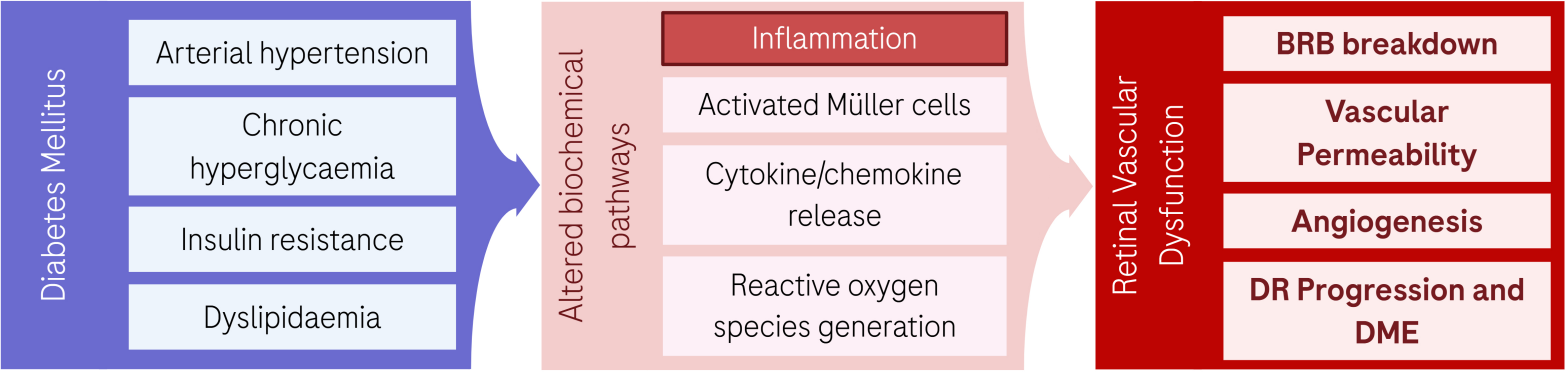
Inflammatory effects of diabetes mellitus in the retina. Chronic hyperglycemia, insulin resistance, arterial hypertension and dyslipidemia can alter multiple biochemical pathways resulting in tissue inflammation, with activated microglia and Müller cells, cytokine/chemokine release, and reactive oxygen species generation. This causes profound retinal vascular dysfunction that manifests as breakdown of the blood-retinal barrier, increased vascular permeability, and angiogenesis, leading to DR disease progression. Created in BioRender. Harlow, R. E. A. (2026). https://BioRender.com/7j77u26.

To contextualize the role of IL-6, it is first necessary to summarize the inflammatory and vascular pathways driving DR. The prolonged exposure to hyperglycemia causes changes in biochemical signaling pathways that lead to loss of homeostasis and produce significant perturbations in the retina (**Figure 2**). Hyperglycemia provokes alterations in the polyol, hexosamine, and protein kinase C (PKC) signaling pathways that lead to aberrant generation of reactive oxygen species (ROS) and mitochondrial dysfunction (21). In turn, mitochondrial impairment triggers additional ROS production causing DNA damage and alterations in protein and lipid structures. Those biochemical disturbances cause tissue injury and inflammation, retinal ganglion cell (RGC) death via apoptosis, and activation of Müller glial cells (22). These cells, in addition to retinal pigment epithelial (RPE) cells and resident microglia, contribute to amplify the inflammation cascade and the subsequent release of VEGF-A and multiple proinflammatory cytokines and chemokines including interleukin-1 beta (IL-1β), tumor necrosis factor alpha (TNF-α), IL-6, interleukin-8 (IL-8), and monocyte chemoattractant protein-1 (MCP-1) (23). This complex milieu of dysregulated proinflammatory factors in the diabetic retina induces changes to the vascular endothelium, promoting leukocyte adhesion and leukostasis (24), which can lead to capillary occlusion, tissue ischemia and vascular damage, which constitute hallmarks of DR. Additionally, dysregulated IL-6 signaling causes tight junction disorganization (25) and BRB breakdown. This provokes macular edema (3) and inflammatory cell infiltration (26), unleashing the inflammatory cascade. Resulting structural changes include increased vascular permeability and tissue remodeling (27), characterized by basement membrane thickening, pericyte loss, and capillary dropout, which can lead to tissue hypoxia and retinal ischemia, and disease progression to proliferative states.

**Figure 3:**
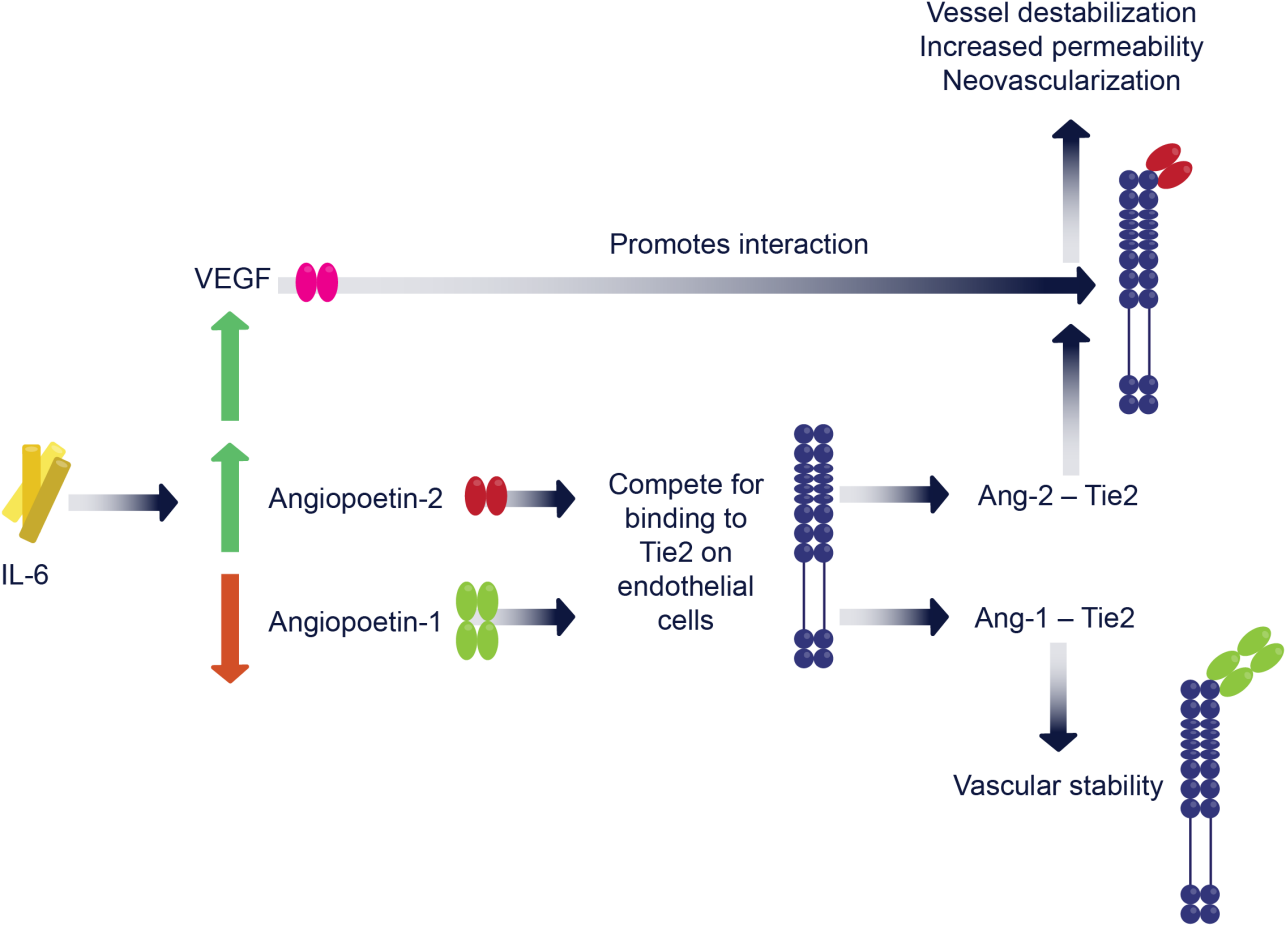
IL-6/VEGF/Ang-2 interplay and cross-talk. Interleukin-6 (IL-6) initiates a cascade by upregulating vascular endothelial growth factor (VEGF) and angiopoietin-2 (Ang-2), while concurrently downregulating angiopoietin-1 (Ang-1). Ang-1 (green) and Ang-2 (red) compete for binding to the Tie2 receptor (dark blue) on endothelial cells. Ang-1 binding promotes vascular stability. Conversely, Ang-2 binding to Tie2, often in conjunction with elevated VEGF levels, leads to vessel destabilization, increased vascular permeability, and pathological neovascularization.

Other key factors in DR pathogenesis include VEGF and Angiopoietin-2 (Ang-2) (**Figure 3**). VEGF has long been recognized as a central driver in diabetic retinal disease pathogenesis (28, 29). VEGF-A, a key isoform of the VEGF family, is produced by a plethora of cell types including Müller glia, endothelial cells, RPE, and astrocytes (30). VEGF is upregulated in hyperglycemic and hypoxic states and its expression can also be induced by IL-6 (31). VEGF acts on endothelial cells inducing vascular permeability and promoting angiogenesis and neovascularization (29), which are characteristic features of proliferative diabetic retinopathy (PDR). However, the newly formed blood vessels are fragile and prone to leakage, resulting in retinal hemorrhages and increased risk of vitreous hemorrhage. VEGF levels are elevated in intraocular fluids of patients with DR (32) and may be correlated with disease severity and progression (33).

Angiopoietin-2 (Ang-2) is a key regulator of vascular remodeling and works in concert with VEGF to destabilize blood vessels via modulation of the balance between pro-angiogenic and anti-angiogenic signals (34). Ang-2 competes with angiopoietin-1 (Ang-1) for binding to the Tie2 receptor on endothelial cells. Ang-1–Tie2 binding promotes vessel stability, whereas Ang-2 in the presence of high VEGF levels leads to vessel destabilization, increased vascular permeability, and neovascularization. IL-6 drives retinal vascular instability through precise molecular mechanisms that directly link its signaling pathways to the modulation of these angiogenic factors. Specifically, this cross-talk is primarily mediated by the activation of the Janus kinase (JAK) and signal transducer and activator of transcription 3 (STAT3) intracellular signaling cascade, which can be triggered by both cis- and trans-signaling routes (35, 36). Upon IL-6 receptor engagement and subsequent gp130 dimerization, STAT3 is phosphorylated and translocates to the nucleus, where it functions as a transcription factor (37). Experimental evidence demonstrates that this IL-6-induced STAT3 activation directly induces the expression and upregulation of VEGF (38). Furthermore, this same STAT3-dependent axis is responsible for a shift in the angiopoietin system, promoting the downregulation of the stabilizing factor Ang-1 while simultaneously increasing the expression of the destabilizing factor Ang-2 (39, 40). Both *in vitro* and *in vivo* models confirm that targeted blockade of the IL-6/STAT3 pathway successfully suppresses this VEGF production, restores tight junction integrity (specifically zona occludens-1 and occludin), and inhibits subsequent neovascularization and vascular leakage (19, 41). Together, this establishes the mechanistic foundation for IL-6 as a critical upstream driver of vascular permeability in diabetic retinal disease.

## 3 Overview of IL-6 biology

**Figure 4:**
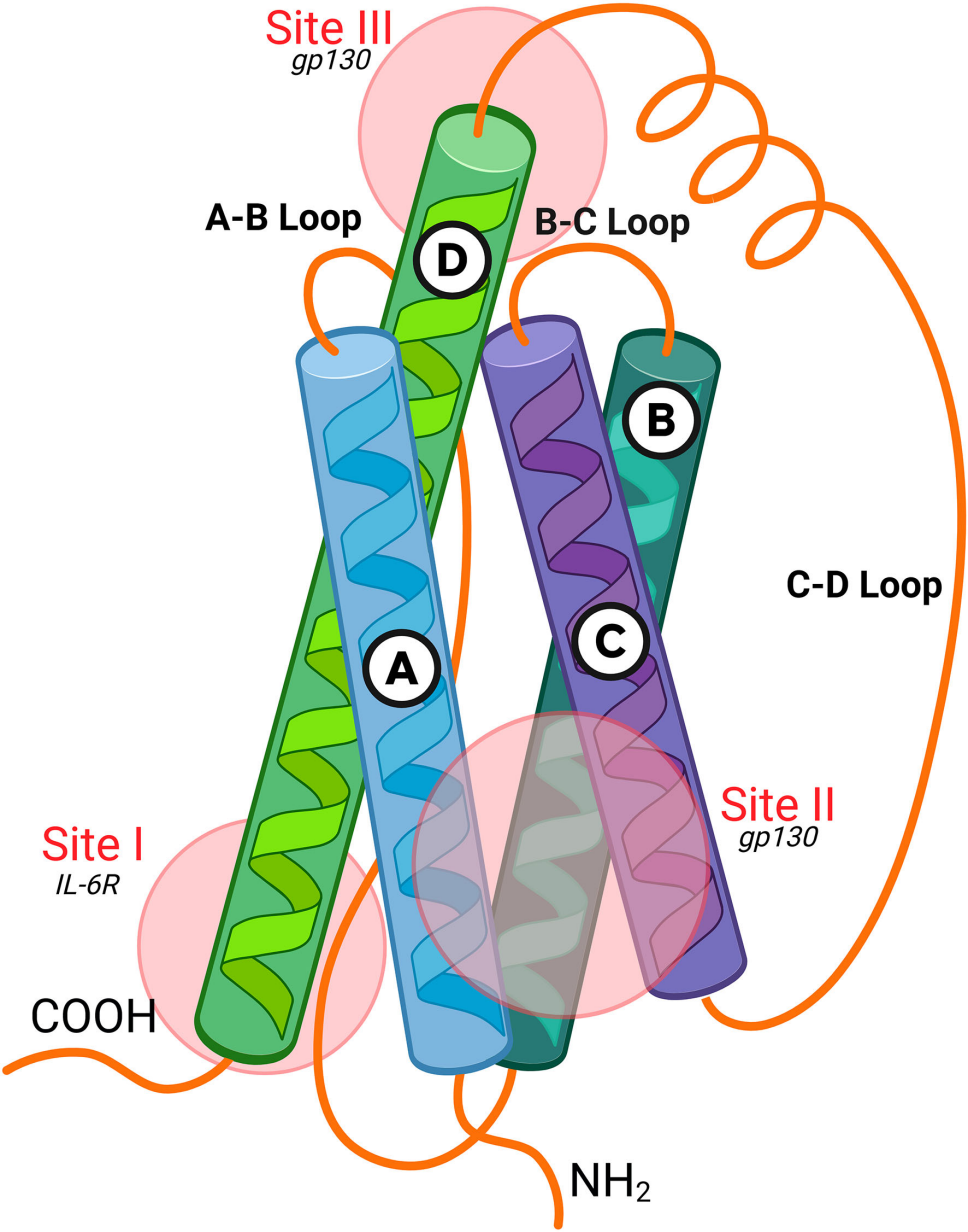
IL-6 protein structure and binding interfaces. The Interleukin-6 (IL-6) cytokine adopts a topology consisting of four antiparallel alpha-helices **(A–D)** connected by loops. The figure highlights three distinct surface regions essential for receptor engagement and subsequent signaling. signaling is initiated sequentially: IL-6 first binds to its specific alpha receptor subunit, IL-6R, via Site I. The resulting complex then recruits two molecules of the signal-transducing subunit, glycoprotein 130 (gp130), which bind to Site II and Site III, respectively. The simultaneous engagement of Site II and Site III is crucial for inducing gp130 homodimerization, the necessary step to activate associated Janus kinases (JAKs) and trigger downstream intracellular cascades, such as the STAT3 pathway. Created in BioRender. Harlow, R. E. A. (2026). https://BioRender.com/kitk002.

**Figure 5:**
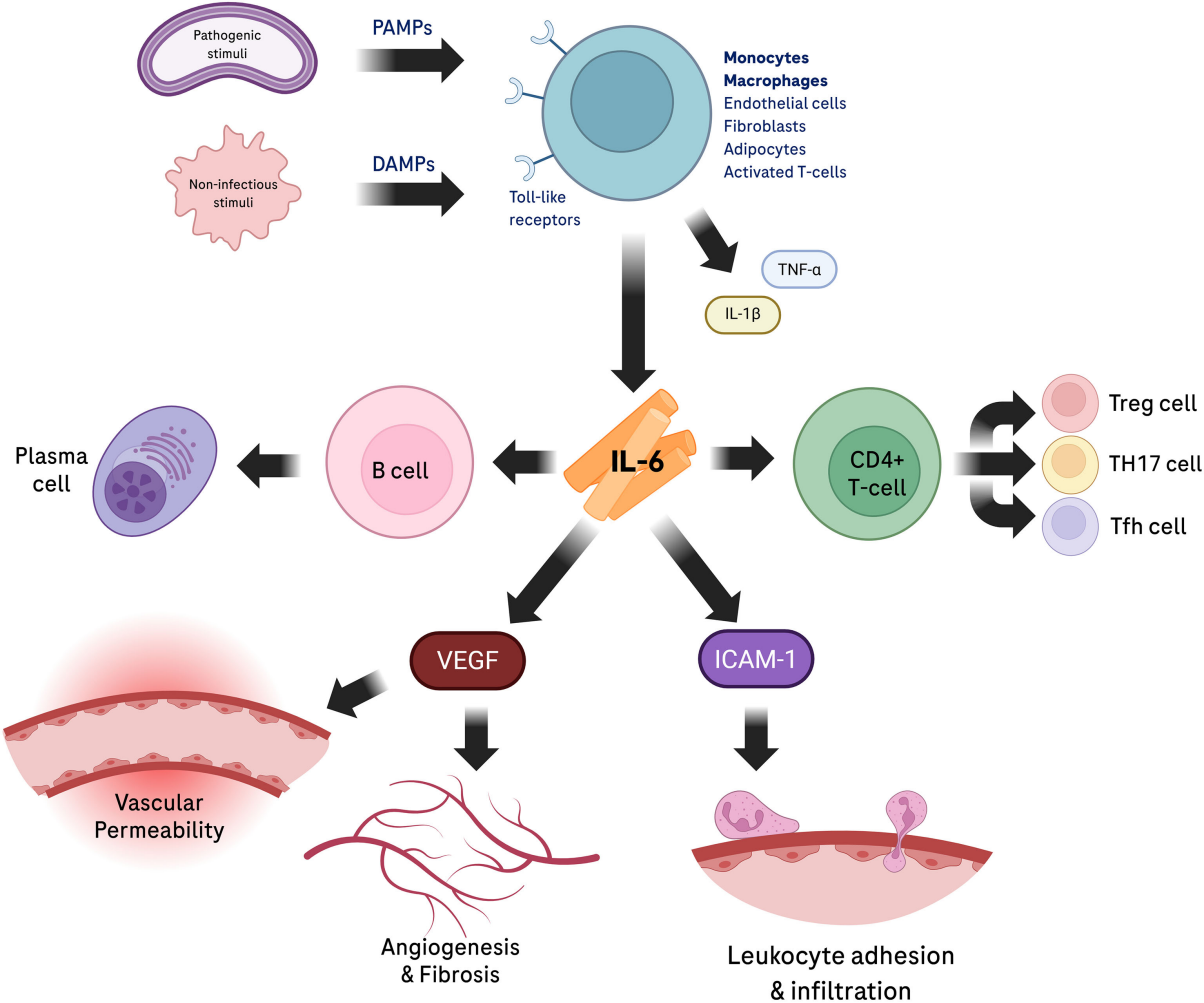
Inflammatory pathways in diabetic retinal disease. Interleukin-6 (IL-6) is produced by various cell types in response to both pathogenic and non-infectious stimuli (danger-associated molecular patterns [DAMPs] and pathogen-associated molecular patterns [PAMPs]) that activate toll-like receptors, leading to immune cell recruitment via upregulation of leukocyte adhesion molecules and facilitating B- and T-cell differentiation, including plasma cell formation. Furthermore, IL-6 enhances the infiltration of immune cells, such as monocytes/macrophages and T cells, by promoting the expression of adhesion molecules on endothelial cells, alongside chemokines released during injury or stress. ICAM-1, intercellular adhesion molecule 1; IL-1β, interleukin-1 beta; Tfh, T follicular helper; TH17, T helper 17; TNF- α, tumor necrosis factor alpha; Treg, T-regulatory; VEGF, vascular endothelial growth factor. Created in BioRender. Harlow, R. E. A. (2026). https://BioRender.com/8z3uodq.

IL-6 is a soluble, four-helical, secreted protein of 184 amino acids and 26kDa encoded by a gene located in chromosome 7p21 (**Figure 4**). Discovered in 1986 by the group of Tadamitsu Kishimoto in Japan (42), it was originally described as a B-cell growth factor, an initiator of acute phase responses, and a mediator of T-cell survival (43). It is produced by various cell types, including T cells, B cells, monocytes, macrophages, fibroblasts, and endothelial cells (44). IL-6 has broad biological activities in multiple target cells, including acute-phase protein synthesis and regulation of various components of the immune system (**Figure 5**).

IL-6 is a pleiotropic cytokine central to systemic defense against infection and tissue injury (**Table 2**). Under physiological conditions, IL-6 is found in trace concentrations in the blood (1-5pg/mL), however during inflammation its levels can increase by more than 100,000-fold (72, 73). IL-6 is expressed in high concentrations at the sites of inflammation and is involved in the pathogenesis of multiple systemic autoimmune diseases and even cancers (74). During infection, monocytes and macrophages rapidly produce IL-6 after activation of Toll-like receptors (TLRs) (including TLR3, TLR4, TLR9) by pathogen-associated molecular patterns (PAMPs) (72, 75). In non-infectious inflammation e.g. tissue stress, damage-associated molecular patterns (DAMPs) stimulate TLRs to activate the IL-6 intracellular signaling cascade (76).The local encounter of innate immune cells with danger signals in early stages of the immune response is translated into systemic dissemination of IL-6 through the bloodstream and rapid elevation of IL-6 levels. Liver hepatocytes respond to the IL-6 stimulus, inducing the synthesis of acute-phase reactants such as C-reactive protein (CRP), serum amyloid A, fibrinogen, and haptoglobin (47). Additionally, IL-6 contributes to the adaptive immune response by acting on B and T lymphocytes, inducing B cell differentiation into immunoglobulin-producing plasma cells (73). Moreover, IL-6 has been implicated in the polarization of specific CD4+ T cell subsets. It is responsible for the balance between Th1 and Th2 effector functions, inhibiting Th1 and promoting Th2 differentiation. Notably, in combination with transforming growth factor β (TGF-β), IL-6 induces the differentiation of the Th17 cell subset, which plays a central role in multiple inflammatory and autoimmune conditions (77, 78). IL-6 is also recognized as a regulator of the balance between pro-inflammatory Th17 and anti-inflammatory T-regulatory (Treg) cells, inhibiting Treg differentiation induced by TGF-β, resulting in a Th17/Treg imbalance that leads to the breakdown of the immunological tolerance, favoring a pro-inflammatory environment (79). Beyond these subsets, IL-6 is also implicated in the differentiation of T follicular helper (Tfh) cells, which are essential for supporting germinal centers and generating memory B cells (80). Additionally, IL-6 can induce the activation of naïve CD8+ T cells, allowing them to acquire cytotoxic functions (81). In summary, due to this vast pleiotropic activity and its major immunomodulatory functions, IL-6 is recognized today as one of the most important cytokines controlling human health and disease.

**Table 2:** The systemic and ocular pleiotropic functions of IL-6.

Pathophysiological system	IL-6 mediated effects & mechanisms	Clinical implications	References
Systemic & Hepatic	Acute-Phase Response: Stimulates hepatocytes to produce acute-phase proteins (e.g., C-reactive protein, Serum Amyloid A).	Systemic inflammation, Atherosclerosis, Cardiovascular disease	Devaraj et al., 2011 (45), Eriksson et al., 1995 (46), Heinrich et al., 1990 (47), Jensen and Whitehead, 1998 (48), Liuzzi et al., 2005 (49), Nemeth et al., 2004 (50)
	Metabolic Regulation: Inhibits the production of albumin and transferrin.	Edema, Anemia of chronic inflammation	Danielski et al., 2003 (51), Weiler et al., 2015 (52)
Hematopoietic	Thrombopoiesis: Stimulates the maturation of megakaryocytes within the bone marrow.	Thrombocytosis associated with inflammation	Ishibashi et al., 1989 (53)
Immune Regulation	T-Cell Differentiation: In concert with TGF-β, it promotes the differentiation of naïve CD4+ T-cells into pro-inflammatory Th17 cells while inhibiting the generation of anti-inflammatory Treg cells.	Th17/Treg imbalance, Autoimmune diseases (including non-infectious uveitis)	Carbone et al., 2013 (54), Fujimoto et al., 2008 (55), Korn et al., 2009 (56)
	B-Cell Maturation: Induces the final differentiation of B-cells into plasma cells that produce antibodies.	Hypergammaglobulinemia, Multiple myeloma	Jego et al., 2004 (57)
Ocular & Retinal Pathophysiology	Vascular Permeability & BRB Breakdown: Upregulates VEGF expression; disrupts endothelial tight junctions (e.g., ZO-1) in retinal endothelial cells and RPE.	Blood-retinal barrier (BRB) breakdown, Macular edema (DME)	Alsaffar et al., 2018 (58), Mesquida et al., 2019 (59), Nagineni et al., 2012 (60), Sharma, 2021 (61), Valle et al., 2019 (18), Yang et al., 2023 (62), Yun et al., 2017 (19)
	Inflammatory Cell Recruitment: Upregulates adhesion molecules (e.g., ICAM-1) on endothelial cells, which promotes the infiltration of leukocytes into retinal tissue.	Leukostasis, Amplification of retinal inflammation	Kimura and Kishimoto, 2010 (63), McLoughlin et al., 2005 (20), Robinson et al., 2021 (64), Wang et al., 2017 (65)
	Vascular Destabilization: Modulates the angiopoietin system by downregulating the stabilizing factor Ang-1 and upregulating the destabilizing factor Ang-2.	Neovascularization, Vascular instability	Joussen et al., 2021 (34), Kayakabe et al., 2012 (39)
	Fibrosis & Cellular Proliferation: Promotes the proliferation and epithelial-mesenchymal transition (EMT) of retinal pigment epithelium (RPE) cells.	Proliferative vitreoretinopathy (PVR)	Chen et al., 2020 (66)
	Neuro-glial Regulation: Exerts dual effects on retinal neurons, providing neuroprotection for photoreceptors while also contributing to the structural degeneration of RGC axons.	Complex role in retinal neurodegeneration and protection	Chidlow et al., 2012 (67), Chong et al., 2008 (68), Glass et al., 2023 (69), Sappington et al., 2006 (70)
Molecular Cross-talk	Promiscuous Receptor Binding: In addition to its own receptor, IL-6 can bind to and signal via the Interleukin-11 receptor (IL-11R).	Potential for pro-fibrotic effects via the IL-11 pathway; suggests that targeting the IL-6 cytokine may be superior to targeting only the IL-6R.	Rose‐John and Jones, 2025 (71)

## 4 IL-6 signaling pathways

**Figure 6:**
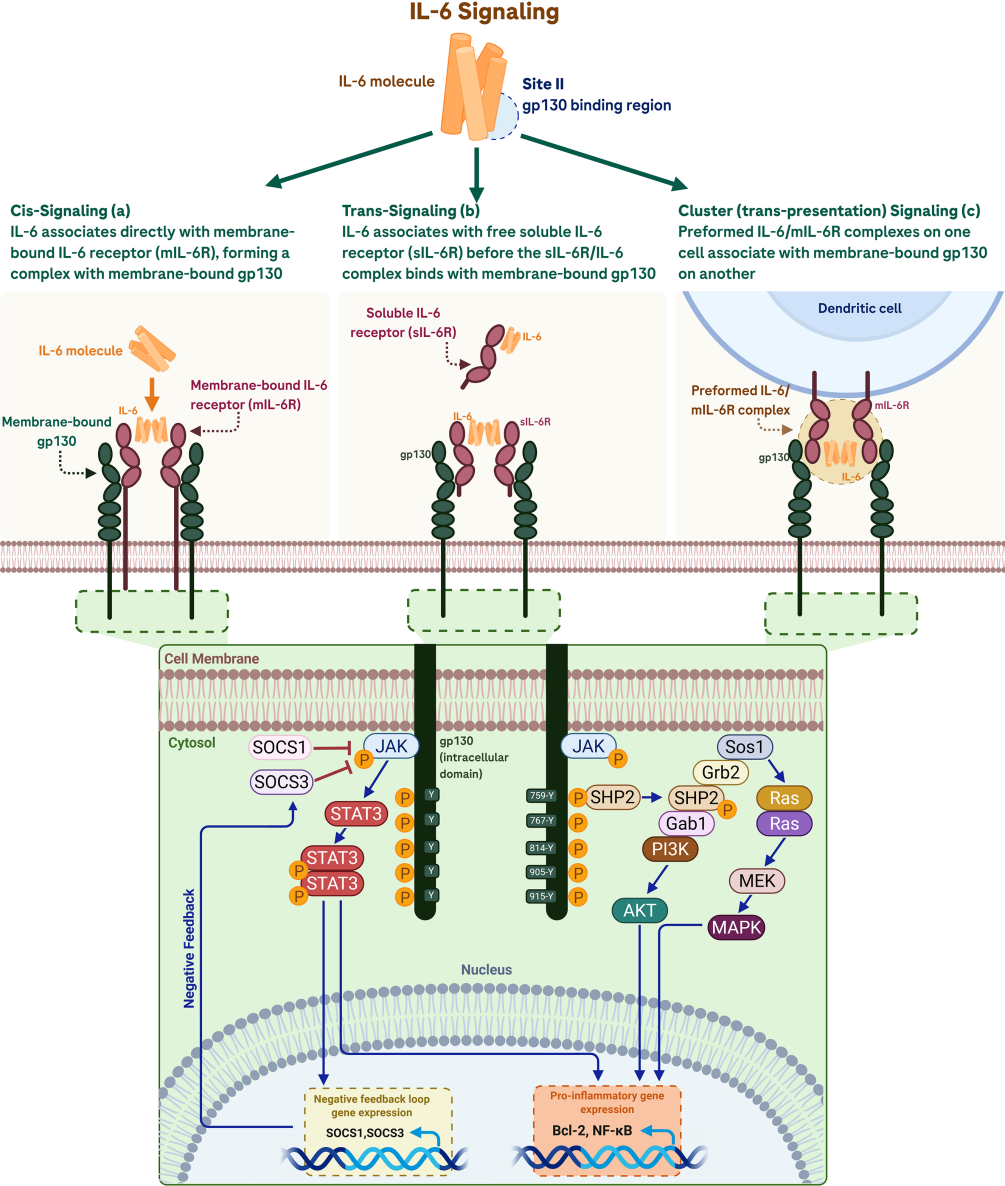
The IL-6 signaling pathways: Cis-, trans-, and cluster (trans-presentation) **(a)** Cis-signaling occurs when IL-6 binds directly to membrane-bound IL-6 receptor (mIL-6R) on the same cell. **(b)** Trans-signaling utilizes soluble IL-6R (sIL-6R) to form a complex with IL-6, which then activates membrane-bound glycoprotein 130 (gp130). **(c)** Cluster signaling (trans-presentation) involves the presentation of preformed IL-6/mIL-6R complexes on a donor cell (such as a dendritic cell) to gp130 on a recipient cell. All three modes trigger gp130 dimerization and initiate downstream intracellular signaling, including the JAK-STAT, Ras-MAPK, and PI3K-AKT pathways. These cascades lead to pro-inflammatory gene expression (e.g., Bcl-2, NF-κB) and the activation of negative feedback loops via SOCS1 and SOCS3. Created in BioRender. Harlow, R. E. A. (2026). https://BioRender.com/idq49cd.

Current knowledge suggests that there are three different modes of IL-6 signaling: cis- (classical) signaling, trans- signaling, and cluster signaling (also known as trans-presentation) (**Figure 6**) (82).

### 4.1 Cis-signaling

The cis- (or classical) pathway was the first recognized mode of IL-6 signaling. Here, IL-6 binds to a transmembrane receptor protein on target cells: the transmembrane IL-6 receptor (mIL-6R). This type of signaling only occurs in cells expressing mIL-6R including hepatocytes and certain leukocyte subpopulations (monocytes, neutrophils, T cells, and B cells) (83). To elicit intracellular signaling, a second receptor protein known as glycoprotein 130 (gp130; the transmembrane signal-transducing protein receptor) is required (82, 84). The association of IL-6/IL-6R to the co-receptor gp130 then leads to the formation of the high affinity activated IL-6/IL-6R/gp130 complex, adopting a hexameric structure consisting of two molecules each of IL-6, IL-6R and gp130. This triggers initiation of the intracellular signal transduction via activation of Janus kinase (JAK) and signal transducer and activator of transcription 3 (STAT3) as well as the JAK-SHP2-Ras-mitogen activated protein kinase (MAPK) pathways, eliciting the downstream signal cascade leading to specific changes in intra-nuclear gene expression of various IL-6-responsive genes (82).

### 4.2 Trans-signaling

Trans- signaling, a mechanism first described by Rose-John and Heinrich (85) extends IL-6’s effects to a broader range of cells that do not express mIL-6R. This pathway involves the formation of a complex between IL-6 and the soluble IL-6 receptor (sIL-6R). This soluble receptor can be generated by two different mechanisms: limited proteolysis of the membrane-bound receptor by the metalloprotease ADAM-17, and by translation of a differentially spliced mRNA. It is thought that regulated generation of sIL-6R occurs predominantly through shedding rather than through differential splicing. In trans-signaling, cells that do not express mIL-6R can be stimulated by the complex of IL-6/sIL-6R and be therefore responsive to IL-6. The IL-6/sIL-6R complex binds to gp130, leading to gp130 dimerization and activation of the downstream cascade. Therefore, using trans-pathway, the IL-6/sIL-6R complex can bind to gp130 on any cell surface, effectively rendering the entire body responsive to IL-6 in the absence of mIL6-R.

However, this potent pathway is strictly regulated by soluble gp130 (sgp130), which circulates in high concentrations in plasma or serum, predominantly falling within the range of 250 to 400 ng/mL (~2.5 to 4.0 nM or ~1.25 to 2.0 nM as the functional homodimer *in vivo*) in healthy individuals (82, 83, 86). In non-inflammatory aqueous humor samples, concentration of sgp130 has been reported at a median of 5.2 ng/mL (~51.5 - 52.0 pM or 25.7 - 26.0 pM as the functional homodimer) (87).Crucially, sgp130 does not bind to free IL-6 or free sIL-6R. It exclusively recognizes and binds the IL-6/sIL-6R complex. By doing so, sgp130 acts as a decoy receptor or molecular trap, competing with membrane-bound gp130. In healthy organisms, sgp130 concentration in plasma/serum vastly outweighs sIL-6R concentrations (~40–75 ng/mL or ~0.8-1.5 nM) (88), ensuring that trans-signaling is neutralized. In disease, such as diabetic retinal disease, this buffering system is overwhelmed. Elevated IL-6 creates a surplus of IL-6/sIL-6R complexes that saturate the available sgp130. The remaining “unbuffered” complexes are then free to bind to cells, e.g. retinal endothelial cells, triggering the pro-inflammatory and barrier-disrupting pathology.

As in cis- signaling, trans- signaling activates the JAK-STAT and Ras-MAPK pathways, influencing gene expression related to inflammation, cell survival, and proliferation (89). This type of signaling is biologically significant because it can lead to a massive expansion of the spectrum of IL-6–responsive cells. Because all cells in the human body express gp130, theoretically any cell may respond to IL-6 in the presence of sIL-6R (90). Moreover, *in vitro* and animal studies suggest that trans- signaling could be responsible for the proinflammatory activities of IL-6 (91, 92).

Trans-signaling is a crucial mechanism in various chronic inflammatory and autoimmune conditions, as it can disseminate inflammation across diverse tissue types. Its involvement is well-documented in diseases such as rheumatoid arthritis (83), atherosclerosis (93), Crohn’s disease (94), and in ocular diseases such as DR/DME (61) and non-infectious uveitis and uveitic macular edema (95, 96).

### 4.3 Cluster signaling

Cluster (or trans-presentation) signaling involves direct cell-to-cell interactions and was discovered in more recent history than the other two signaling modalities (97). In this pathway, preformed complexes of IL-6 and mIL-6R on one cell surface (e.g., that of dendritic cells) activate gp130 on an adjacent target cell. IL-6 and mIL-6R form complexes on transmitter cells, which engage gp130 on neighboring receiver cells. The close cell-to-cell contact in trans-presentation ensures signaling is restricted to adjacent target cells and prevents widespread and uncontrolled activation (98). Cluster signaling is involved in the priming of pathogenic Th17 cells (97, 98) and is particularly relevant in the context of autoimmune diseases.

All three signaling modalities ultimate in the same intracellular downstream signaling pathway, triggered by the dimerization of gp130. However, integral to these signaling cascades is a self-regulatory negative feedback loop. One of the first genes induced after STAT activation is the suppressor of cytokine signaling 3 (SOCS3), a JAK feedback inhibitor, which ultimately leads to termination of the IL-6 signaling cascade and arrest of the process (99). In chronic or severe inflammatory disease states, despite activation of SOCS3, the overwhelming influence of IL-6 overrides this feedback mechanism, thus resulting in uncontrolled inflammation (44, 100, 101).

### 4.4 The IL-6 buffer system

**Figure 7:**
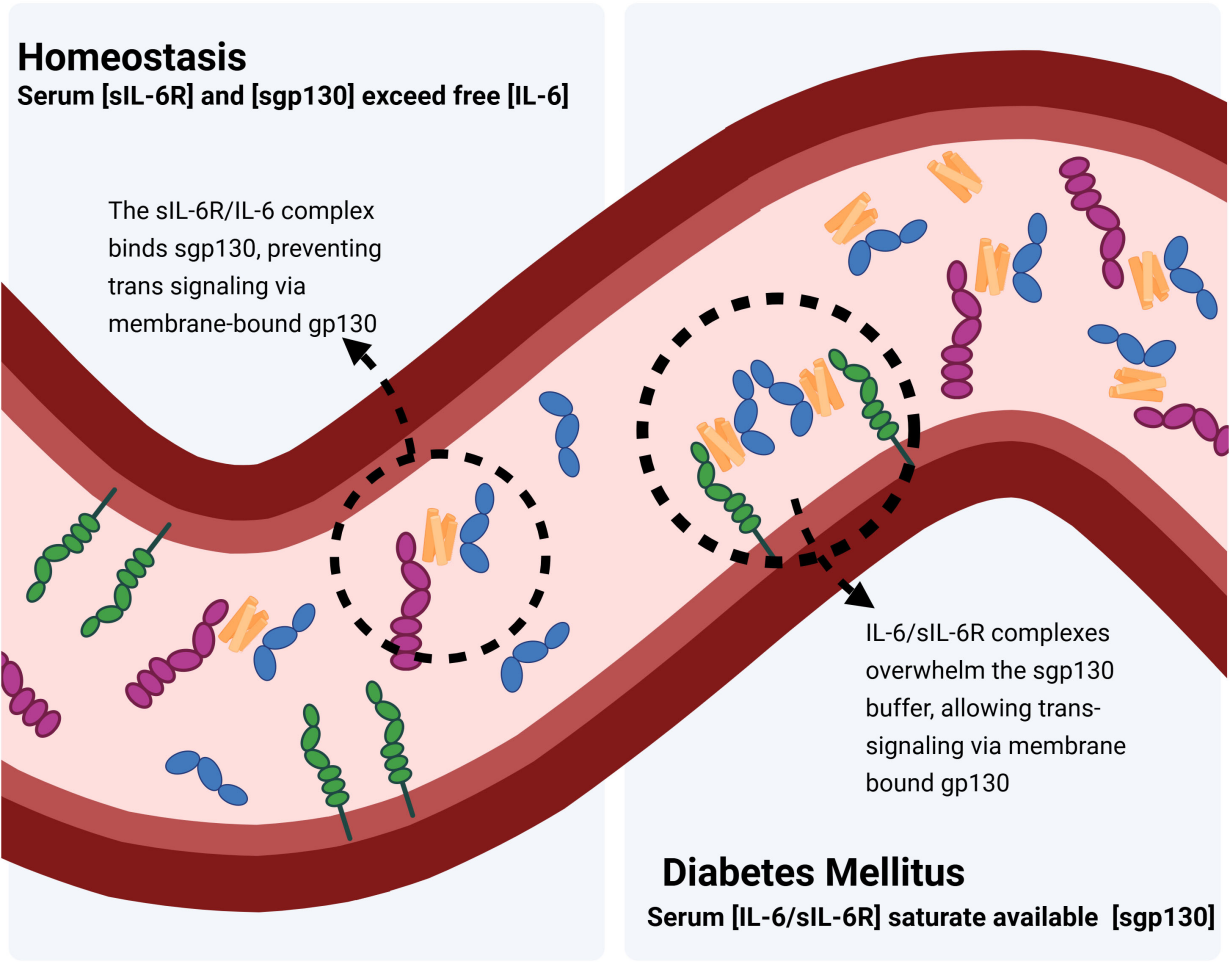
The IL-6 buffer system in homeostatic and diabetic states. In healthy individuals (left), sufficient soluble glycoprotein 130 (sgp130, purple) exceeds free IL-6 (orange), allowing sgp130 to bind IL-6/soluble IL-6 receptor (sIL-6R, blue) complexes and prevent trans- signaling via membrane-bound gp130 (green). Conversely, in diabetes (right), elevated IL-6 levels bind available IL-6R and overwhelm the sgp130 buffer, leading to unchecked trans- signaling and dysregulated inflammation. Created in BioRender. Harlow, R. E. A. (2026). https://BioRender.com/04rljv7.

As aforementioned, IL-6 serum concentrations in healthy individuals are very low, around 1–5 pg/mL (~0.05 to 0.25 pM) in serum/plasma, and around 0.3-3.8 pg/mL (~0.015 to 0.19 pM) in aqueous humor cataract controls (83, 102, 103). In contrast, serum concentrations of sIL-6R and sgp130 are much higher, in the range of 40-80 ng/mL and 250–400 ng/mL (~0.82-1.36 nM), respectively in plasma/serum (82, 83, 86), and 36.5 pg/mL (~0.66 pM) and 5.2 ng/mL (~51.5 - 52.0 pM or 25.7 - 26.0 pM as the functional homodimer) in the aqueous humor of non-inflammatory controls (87, 104)During inflammatory states, the levels of sgp130 are largely maintained, whereas levels of sIL-6R increase by a factor of 2-5-fold. In sepsis for instance, IL-6 concentrations can even rise to the μg/mL range. IL-6 binds to mIL-6R with a low affinity of approximately 1nM. However, the resulting complex (IL-6/IL-6R) associates with the signal transducer gp130 with 100-fold higher affinity of 10 pM (105). Therefore IL-6 secreted into the bloodstream will bind to soluble IL-6R, and the complex of IL-6/sIL-6R will immediately bind to sgp130 and will subsequently be neutralized. In consequence, the high amounts of sIL-6R and sgp130 in serum constitute a buffer for IL-6 since the concentration of sgp130 vastly exceeds the concentration of the sIL-6R, which is indeed the limiting factor for the capacity of the IL-6 buffer in the blood (106) (**Figure 7**). In various systemic inflammatory conditions including diabetes mellitus, the IL-6 buffer system is often disturbed, leading to dysregulated IL-6 signaling (107). Interestingly, it has been shown that individuals with the single nucleotide polymorphism (SNP) rs2228145 (which leads to a change close to the ADAM-17 cleavage site for IL-6R) have significantly higher sIL-6R levels in serum. These higher IL-6R levels lead to a higher capacity of the IL-6 buffer in the blood, which has an anti-inflammatory effect and is protective against cardiovascular and autoimmune disease (108).

## 5 Preclinical evidence for IL-6 in diabetic retinal disease

Mounting evidence points to the pathogenic contribution of IL-6 in DR/DME (15, 69, 109, 110). The IL-6 signaling pathway is integral in the pathogenesis of DR and other inflammatory eye diseases due to its involvement in endothelial cell dysfunction and vascular inflammation. Several lines of evidence suggest that IL-6 is a crucial mediator of both VEGF-mediated and inflammatory vascular leakage, through either direct or indirect effects on the vascular endothelium. While classical signaling is largely restricted to immune cells, trans-signaling allows IL-6 to act directly on the retinal vasculature, where the mIL-6R is typically absent but gp130 is ubiquitously expressed. Interactions between IL-6 and endothelial cells regulate recruitment of leukocytes and expression of inflammatory proteins. Indeed, IL-6 can promote increased vascular permeability via direct barrier-disrupting effects on endothelial and epithelial cells or by inducing production of other cytokines and growth factors including the pro-permeability and pro-angiogenic factor VEGF (31).

In the preclinical setting, Valle et al. recently reported that IL-6 trans-signaling causes barrier disruption in human retinal microvascular endothelial cells (HRMECs) via upregulation of Intercellular Adhesion Molecule-1 (ICAM-1), an endothelial cell specific leukocyte adhesion molecule which serves as the molecular tether for leukostasis and immune response (18, 64). Their findings suggest that IL-6 trans-signaling causes reactive oxygen species (ROS)-induced damage, mitochondrial dysfunction, inflammation, apoptosis, and the loss of barrier function in HRMECs, and blockade of IL-6 trans-signaling prevents inflammation and endothelial barrier disruption. In another *in vitro* study, Yun et al. demonstrate that IL-6-induced STAT3 activation increases endothelial permeability through the downregulation of Zonula Occludens-1 (ZO-1) and occludin, which are main components of intercellular tight junctions forming the iBRB (19). On the other hand, IL-6 has also been found to promote ocular angiogenesis in different cell lines via induction of VEGF production. Moreover, in the murine laser-induced choroidal neovascularization (CNV) model, antibody-based blockade of the IL-6R or genetic ablation of IL-6 led to a significant suppression of CNV and vascular leakage. CNV generation was accompanied by activation of the transcription factor STAT3 in choroidal endothelial cells and macrophages, and IL-6R blockade resulted in suppression of CNV formation and inhibition of subretinal fibrosis (111). Lastly, IL-6 has been strongly linked to macular edema formation. Our group’s research on the ARPE-19 cell line, modeling the outer BRB, confirmed that IL-6 exposure leads to a reversible decrease in transepithelial electrical resistance and a disruption of ZO-1 localization. The fact that IL-6 receptor blockade with tocilizumab restored barrier integrity provides a strong rationale for targeting the IL-6 pathway to treat macular edema that is refractory to anti-VEGF monotherapy (59).

## 6 Clinical evidence for IL-6 in diabetic retinal disease

From the clinical perspective, intraocular fluid concentrations of IL-6 are frequently elevated in patients with DR and DME (15, 112) where this cytokine has been suggested to orchestrate the underlying pathology.

A recent large-scale prospective study by Manda et al. (15) including 328 eyes, provided evidence that aqueous humor (AH) IL-6 levels are significantly associated with DR severity and disease progression. In this cohort, median AH IL-6 levels progressively increased from eyes with no DR (5.40 pg/mL) to those with moderate non-proliferative DR (9.25 pg/mL) and proliferative DR (15.71 pg/mL). Furthermore, the group identified a significant positive correlation between AH IL-6 concentrations and both central subfield thickness and macular volume, establishing increased IL-6 as a significant predictor for the presence of DME. These authors postulate that due to its relatively short half-life, intraocular IL-6 levels reflect real time biologic activity within the eye and may be more closely correlated with disease activity.

These findings are in line with other case series and meta-analysis which have suggested that aqueous and vitreous concentrations of IL-6 are elevated in patients with DME compared to controls (**Table 3**) (110, 117). The local nature of this inflammation is supported by studies showing that while vitreous and aqueous humor IL-6 levels are elevated in DR patients, serum levels often remain low, implying intraocular production rather than systemic leakage (120, 121). Aqueous humor analyses have also demonstrated significant correlations between IL-6 and other angiogenic factors including VEGF (14, 122), reinforcing the concept that IL-6 is a key component of the inflammatory milieu driving diabetic retinal pathology. Funatsu et al. measured cytokine levels in the aqueous humor of DME patients, and found that higher IL−6 levels were significantly correlated with greater severity of macular edema (112). In addition, a *post-hoc* analysis of the READ3 study in which DME patients were randomized to receive 0.5 or 2.0 mg ranibizumab and AH samples were collected in a prospective manner, has revealed that those patients who presented elevated aqueous humor levels of IL-6 at baseline ended up with worse visual outcomes when compared to those with initial low aqueous IL-6 levels (16).

**Table 3:** IL-6 and VEGF aqueous humor and vitreous levels in diabetic eye disease.

Publication	Population	IL-6 (pg/mL)	VEGF (pg/mL)
Funatsu et al., 2003 (113)	n=26 DME n=12 nondiabetic ocular disease (controls)	DME^a^: 188.1 (18.0–768.4) Controls^a^: 9.51 (4.02–22.4) *P* < 0.0001	DME^a^: 818.0 (36.8–1902.4) Controls^a^: 17.8 (15.6–26.2) *P* < 0.0001
Murugeswari et al., 2008 (114)	n=25 PDR n=25 macular hole (controls)	IL-6 higher in PDR (*P* < 0.0001)	VEGF higher in PDR (*P* < 0.0001)
Funatsu et al., 2009 (115)	n=53 DME n=15 non-diabetic ocular disease (controls)	DME^a^: 192.4 (18.0–823.4) Controls^a^: 8.74 (4.00–23.2) *P* < 0.05	DME^a^: 1086.4 (15.6–3450.0) Controls^a^: 20.4 (15.6–69.6) *P* < 0.05
Wu et al., 2020 (116)	n=26 PDR n=27 non-diabetic ocular disease (controls)	PDR^a^: 157.5 (25.0–1401.0) Controls^a^: 44.0 (5.0–4425) *P* = 0.021	N/A
Minaker et al., 2022 (117)	Meta-analysis of 128 studies (2002–2020) n=4163 DME eyes n=1281 controls	IL-6 higher in AH in DME *P* < 0.0004	VEGF higher in both AH and VH in DME *P* < 0.00001
Mason et al., 2022 (118)	Meta-analysis of 341 studies (1992–2020) n=10379 PDR eyes n=6269 controls	IL-6 higher in PDR *P* < 0.00001	VEGF higher in PDR *P* < 0.00001
Sato et al., 2023 (119)	n=25 PDR n=14 macular hole n=16 ERM (controls)	PDR^b^: 133.2 ± 159.1 Controls^b^: 8.53 ± 19.4 *P* = 0.001	PDR^b^: 870.0 ± 1680.8 Controls^b^: 0.16 ± 0.89 *P* < 0.0001
Manda et al., 2025 (15)	n=328 eyes (46 PDR; 46 no DR controls)	AH - PDR^c^: 15.71 (9.24–48.58); No DR Control^c^: 5.40 (2.99–8.77); *P* < 0.001	N/A

a Median (range).

b Mean ± SD.

AH, aqueous humor; DME, diabetic macular edema; ERM, epiretinal membrane; IL-6, interleukin-6; N/A, not applicable; PDR, proliferative diabetic retinopathy; VEGF, vascular endothelial growth factor; VH, vitreous humor.

Intraocular IL-6 concentrations suggest a potential prognostic role and may point to its utility as a candidate predictive biomarker for suboptimal response to anti-VEGF therapy. Taken together, the clinical evidence supports the hypothesis of IL-6 as both a potential biomarker of disease severity and a therapeutic target. These associations are consistent with the cytokine’s proposed pathogenic contribution and support the development of strategies directed against this pathway.

## 7 Therapeutic implications of targeting IL-6

Therapeutic blockade of IL-6 signaling may be conducted either through inhibition of the IL-6 cytokine itself, or by targeting its receptor. Monoclonal antibodies targeting IL-6R include tocilizumab, sarilumab, and satralizumab, while sirukumab, olokizumab, and clazakizumab target the ligand itself (123). While anti–IL-6 therapeutics have been largely investigated and developed for systemic autoimmune-mediated and inflammatory diseases, there has been a growing expansion into cardiovascular and metabolic conditions, as well as the recent development efforts in ocular disease such as neuromyelitis optica (124) and thyroid eye disease (TED) with satralizumab (125).

From a retinal disease perspective, the identification of IL-6 as a critical mediator of retinal inflammation and vascular permeability has driven the development of novel agents such as vamikibart and bispecific antibodies such as RO7497372 (126). Vamikibart is a recombinant, humanized monoclonal antibody engineered for intravitreal administration that binds to and inhibits IL-6 activity. The clinical efficacy and safety of vamikibart have been evaluated through a sequential trial program involving patients with DME and Uveitic Macular Edema (UME). The Phase I DOVETAIL study (NCT06771271) investigated the safety, tolerability, pharmacokinetics and pharmacodynamics of vamikibart as both a monotherapy and in combination with ranibizumab in participants with DME and UME. Results from this early-phase trial indicated that vamikibart was well tolerated and preliminary signs of efficacy were observed (127–129). These positive outcomes supported further clinical development of vamikibart in both DME and UME participants. In the context of DME, two Phase II studies were initiated. ALLUVIUM (NCT05151731) was designed to investigate the efficacy and safety of vamikibart monotherapy compared to ranibizumab. On the other hand, BARDENAS (NCT05151744) evaluated vamikibart in combination with ranibizumab versus ranibizumab monotherapy. These two Phase II studies have now been completed, and results will be communicated soon. Concurrently, vamikibart has progressed to global registrational Phase III trials in UME: MEERKAT (NCT05642312) and SANDCAT (NCT05642325). these two identical, randomized, double-masked, sham-controlled trials evaluated the efficacy of intravitreal vamikibart in macular edema associated with non-infectious uveitis (NIU).

A significant clinical “pain point” in treating patients with DME is the trade-off required when using steroids. While steroids are powerful anti-inflammatories, their lack of specificity leads to off-target effects. A primary therapeutic advantage of targeting IL-6 specifically is the potential to decouple anti-inflammatory efficacy from the adverse ocular effects associated with broad-spectrum corticosteroid therapy (130). Local corticosteroids are regarded as second-line therapy to treat refractory DME, and are well-known to carry significant risks, including elevation of intraocular pressure (IOP), development of glaucoma, and cataract formation.

The transition from monotherapy to bispecific platforms in patients with diabetic retinal disease represents a shift toward addressing the “multifactorial” escape mechanisms seen in the incomplete anti-VEGF non-responders. While vamikibart addresses the IL-6 pathway specifically, bispecific agents like RO7497372 (anti-IL-6/VEGF) are designed to neutralize the synergistic loop between inflammation and angiogenesis. RO7497372 is a bispecific antibody that simultaneously targets both IL-6 and VEGF which is currently being investigated in the Phase I PREGONDA study (NCT06847854), which has been initiated to evaluate the safety, tolerability, pharmacokinetics and pharmacodynamics of this novel therapeutic in patients with DME (126). By tackling inflammation, vascular permeability, and angiogenesis, the dual inhibition of VEGF and IL-6 may yield differentiated outcomes in vision and anatomy in this disease context.

One potential future application of IL-6 targeting could be explored within a precision medicine approach. Preliminary clinical evidence (16) has suggested that patients with high baseline intraocular IL-6 might have poorer outcomes with ranibizumab monotherapy. This concept opens the possibility of moving beyond the “VEGF-first” trial-and-error approach, with exploratory identification of biomarkers representative of “inflammatory phenotype”. Hypothetically, future management might investigate prioritizing early intervention with dual IL-6/VEGF inhibition, with the potential to prevent structural damage and capillary dropout by addressing the IL-6 pathway, a recognized player in pathophysiology in patients with diabetic retinal disease.

## 8 Conclusions

Diabetic retinal disease is a global public health problem and a leading cause of blindness. Therapies such as anti-VEGF have been a major breakthrough in the management of vision-threatening stages of DR and are considered first line therapy for DME. While these treatments address angiogenesis and vascular permeability, they remain insufficient for a notable proportion of patients who exhibit suboptimal responses or require burdensome injection frequencies. This “ceiling of efficacy” reached by a significant portion of patients represents a tremendous opportunity for improving outcomes for diabetic patients. This review underscores that while VEGF is a primary driver of vascular permeability and angiogenesis, it is the chronic, low-grade inflammation largely orchestrated via IL-6 signaling, that may be responsible for sustained disease activity in suboptimal responders.

Experimental evidence highlights IL-6 as an upstream driver of retinal inflammation and vascular destabilization. IL-6 alters the angiogenic balance by upregulating VEGF and shifting the angiopoietin system towards pathological vascular permeability. Furthermore, clinical data demonstrate that elevated intraocular IL-6 levels strongly correlate with the severity of diabetic retinopathy and the presence of macular edema. Collectively, these findings demonstrate that targeting the IL-6 pathway offers a promising and multifaceted therapeutic avenue capable of addressing the complex pathological mechanisms left unresolved by current standard-of-care.

There are a plethora of innovative therapeutic approaches being investigated in clinical trials for the treatment of DME, either in monotherapy or in combination with anti-VEGF agents.

1. Selective inhibition: Moving away from the “collateral damage” of corticosteroids toward targeted biological anti-IL-6 agents, which decouple anti-inflammatory power from the risks of ocular hypertension and cataracts. Therefore, the novel anti-inflammatory biologics may be more suitable for the long-term treatment required for chronic diseases such as DR and DME, albeit more evidence is needed in regard to their safety and efficacy. These aspects will be clarified in well-controlled clinical trials that are already underway.
2. Dual-pathway blockade: Utilizing a bispecific approach to address the IL-6 pathway, as well as its synergistic loop with VEGF, may effectively short-circuit the molecular escape routes underlying incomplete response with exacerbating or persistent edema in patients with DME.

Ultimately, an improved understanding of the inflammatory drivers of DR and DME is essential to evolve current treatment strategies. Moving beyond VEGF-first paradigms and a “one-size fits all” approach, future management may allow prioritization of precision medicine approaches, with the potential to stratify patients by their specific inflammatory phenotype. This shift will enable the selection of targeted therapies such as IL-6 inhibition to ensure optimal intervention for each patient and advancing the standard of care in diabetic retinal disease. Matching the right patient with the right therapy at the right time may offer the opportunity to not only treat diabetic retina disease but to fundamentally alter the progression and prognosis of the disease preserving long-term visual function.
